# Low Tissue Creatine: A Therapeutic Target in Clinical Nutrition

**DOI:** 10.3390/nu14061230

**Published:** 2022-03-15

**Authors:** Sergej M. Ostojic

**Affiliations:** 1 Department of Nutrition and Public Health, University of Agder, Universitetsveien 25, 4604 Kristiansand, Norway; sergej.ostojic@uia.no; Tel.: +47-38-14-13-64; 2 Applied Bioenergetics Lab, Faculty of Sport and PE, University of Novi Sad, 21000 Novi Sad, Serbia

**Keywords:** creatine, magnetic resonance spectroscopy, cost-effectiveness, brain, muscle, nutrition

## Abstract

Low tissue creatine characterizes many conditions, including neurodegenerative, cardiopulmonary, and metabolic diseases, with a magnitude of creatine shortfall often corresponds well to a disorder’s severity. A non-invasive monitoring of tissue metabolism with magnetic resonance spectroscopy (MRS) might be a feasible tool to evaluate suboptimal levels of creatine for both predictive, diagnostic, and therapeutic purposes. This mini review paper summarizes disorders with deficient creatine levels and provides arguments for assessing and employing tissue creatine as a relevant target in clinical nutrition.

## 1. Introduction

Phosphorylated creatine (2-[carbamimidoyl(methyl)amino]acetic acid) is the main high-energy phosphate-storage compound able to promptly regenerate adenosine triphosphate (ATP) by the action of creatine kinase, with ATP being the critical energy-containing molecule found in all living cells. Creatine-phosphocreatine system serves both as a temporal and spatial energy buffer of ATP levels, and intracellular energy transport carrier connecting sites of energy production with sites of energy utilization [1,2]. Humans can obtain creatine via endogenous synthesis (from amino acids glycine and *L*-arginine) and through different omnivorous foods (especially red meat and fish), accounting for a total daily creatine output of approximately 2 g for a 70 kg young adult man [3,4,5]. Creatine homeostasis appears to be finely tuned to the dynamics of its endogenous–exogenous provision and utilization as an energy facilitator. This is illustrated by the relatively stable intracellular levels of total creatine (creatine plus phosphocreatine) across various energy-demanding tissues in normal conditions, including the brain (4–5 mM) [6], skeletal muscle (25–30 mM) [7], and myocardium (25–30 mM) [8]. On the other hand, a reduction in intracellular creatine concentrations could result in a hypo-energetic state that occurs in a broad spectrum of pathophysiological situations, with a degree of creatine deficit often correlates with a disorder severity. This mini review summarizes disorders with low creatine levels and provides arguments for assessing and employing tissue creatine as a relevant target in clinical nutrition.

## 2. Creatine Shortfall in Clinical Medicine

The reduced levels of intracellular creatine complement many inherited and acquired disorders, with cerebral creatine deficiency syndromes (CCDS) often considered a clinical paradigm of creatine shortfall. CCDS are rare inborn errors of creatine synthesis machinery and transport, being autosomal recessive or X-linked conditions [9]. CCDS patients typically show clinical features of low bioenergetics (e.g., muscle hypotonia, speech delay, seizures, intellectual impairment, behavioral abnormalities), with low levels of creatine found in the brain, skeletal muscle, and biofluids considered an essential element of preliminary and confirmatory diagnosis of these conditions [10]. However, CCDS often remain underdiagnosed since the assessment of creatine metabolism is not routinely included in standard diagnostic workup [11]. Other neurological pathologies accompanied by insufficient cerebral creatine levels (or impaired creatine-related metabolic ratios) include autism spectrum disorder (ASD) [12], traumatic brain injury [13], multiple sclerosis [14], gyrate atrophy of the choroid and retina [15], post-viral fatigue syndrome [16], and brain malignancies [17,18], while creatine levels were suboptimal in the skeletal muscle of neurological patients suffering from muscular dystrophy and neuromuscular diseases [19,20]. Several cardiovascular conditions exhibit a decrease in myocardial or subendocardial creatine, such as dilated cardiomyopathy [21], aortic valve disease [22], and transplanted heart [23]. Interestingly, it appears that a reduction in myocardial creatine significantly correlates with the clinical severity of heart failure [24], with tissue creatine put forward as an independent multivariate predictor of cardiovascular mortality in patients with failing hearts [25,26]. Tissue creatine levels were also diminished in the skeletal muscle and brain of patients with chronic respiratory failure [27,28], extracts from lung cancer patients [29], pancreatic parenchyma in patients with adenocarcinoma [30], in the brain of patients with hepatitis C and chronic HIV infection [31,32], and in the thalamus of infants with malnutrition [33], while suboptimal creatine levels are suggested in chronic kidney disease [34], and preterm birth [35]. Although the causes of the above conditions are different, they all share a disease-associated creatine deficit. The lack of creatine might not be necessarily the underlying cause for all conditions listed above but perhaps a possible side effect of these diseases. Nevertheless, patients with lower levels appear to be at a higher risk of developing more severe illness [10,24], implying low tissue creatine as a possible prognostic indicator of disease severity. In addition, it might be used as a predictive biomarker to identify individuals with specific conditions who are more likely to experience a favorable effect from the exposure to supplemental creatine. Cut-off points for low tissue creatine are not established so far for specific organs and pathologies, yet various degrees of deficiency of the cerebral creatine pool are seen in neurology, from almost complete depletion of the cerebral creatine pool in CCDS [36,37] to a partial reduction (~10%) seen in other conditions [38,39] (Table 1).

**Table 1:** Summary of human studies (excluding CCDS) describing low tissue creatine levels.

Pathology	Refs.
Autism spectrum disorder	[40,41,42,43,44,45]
Concussion and mild traumatic brain injury	[13,46]
Multiple sclerosis	[47,48,49,50,51]
Gyrate atrophy of the choroid and retina	[15,52]
Post-viral fatigue syndrome	[53,54]
Primary and secondary brain tumors	[17,18]
Neuromuscular disease	[19]
Facioscapulohumeral muscular dystrophy	[20]
Dilated cardiomyopathy	[21,24,25]
Aortic valve disease	[22]
Heart transplantation	[23]
Coronary disease	[26]
Chronic obstructive pulmonary disease	[27,28]
Lung cancer	[29]
Pancreatic cancer	[30]
Hepatitis C	[31]
Chronic HIV infection	[32]
Infant malnutrition	[33]

## 3. Methods for Tissue Creatine Evaluation

A breakthrough in monitoring creatine levels has occurred with magnetic resonance spectroscopy (MRS), a state-of-the-art analytical technique that can be used to non-invasively measure biochemical changes across different tissues and organs. By observing local magnetic fields around atomic nuclei, MRS allows the measurement of in vivo chemical information in a specific volume of interest, with creatine included in routine MRS profiling [55]. Each metabolite has a different peak in the spectrum, which appears at a known frequency (e.g., creatine at 3.03 ppm). Both proton and non-proton MRS techniques are available, with ^1^H MRS appearing to be more convenient than other methods to evaluate creatine concentrations. More accurate MRS methods of creatine assessment involve absolute quantification of creatine levels instead of resonance amplitudes presented as ratios [56]. The experts’ working group on reporting standards for MRS recently provided a set of rules required for the acquisition, post-processing, and analysis required for appropriate interpretation of results [57], facilitating MRS use in everyday practice. MRS is a relatively expensive technique and requires an educated technician, yet it has gained popularity in recent years, as illustrated by more studies employing MRS in nutritional research [58]. The fact that MRS systems are now available in most medical centers allows the routine evaluation of creatine metabolism in many pathologies, at least those that affect energy-demanding organs. Alternatively, tissue biopsy might provide a means to assess creatine levels in specific tissues (predominantly the skeletal muscle), yet this procedure’s invasiveness and possible complications prevent its ample applicability in regular practice. Possible surrogate indices of tissue creatine, including serum or salivary creatine, liquid biopsy, and creatine in cerebrospinal fluid, require more evidence to support or prove its use as MRS substitutes.

## 4. Transition of Tissue Creatine Evaluation from Research to Routine Practice

A meaningful application of any biomarker holds a considerable value in nutrition and medicine, yet a quest from its recognition to clinical use is often slow and arduous [59]. In the case of tissue creatine quantified by MRS, a number of requirements for a successful transition from the research environment to routine clinical practice appear to be nearing completion, with its possible uses potentially encompassing both predictive and diagnostic purposes. The analytical validity of MRS suggests relatively high reproducibility for tissue metabolites measurements (including total creatine) in various tissues [60,61]. MRS employing creatine-related metabolic ratios has high diagnostic accuracy and clinical validity in differentiating neoplasms from non-neoplastic brain tumors [62], brain metastases and primary high-grade gliomas [63], tremor-dominant Parkinson’s disease (PD) and non-PD tremor [64], various parkinsonian syndromes [65], high-functioning adults with ASD and typically developing peers [66], and bone and soft tissue tumors versus normal muscle [67]. In addition, MRS sensitivity (92%) and specificity (76%) were equivalent to other superior methods in aiding the localization of prostate metabolic abnormalities that might include creatine [68]. Evaluating the clinical utility of tissue creatine is still under critical observation. Early studies suggest that MRS-driven creatine indices can help distinguish responders and non-responders to drug treatment in insomnia patients [69], with the test likely improving healthcare and changing outcomes. Although no extensive cost-effectiveness trials are available at this moment, a study suggests that abnormal creatine levels consistent with myocardial ischemia predicted cardiovascular outcomes, higher rates of anginal hospitalization, repeat catheterization, and more significant treatment costs [26]. Another trial suggests that the use of MRS may be cost-effective in specific contexts, for example, in settings where the cost of transrectal ultrasound-guided prostate biopsy exceeds the cost of obtaining an MRS sequence by ~£115 [68]. The above studies suggest added value and costs saved by knowing a patient’s tissue creatine levels.

## 5. Creatine in Clinical Nutrition

A lack of cellular energy as a cause of disease has recently regained attention [70,71], and tissue creatine shortage might be a suitable proxy that characterizes poor bioenergetics seen in conditions that impact top energy-consuming organs. Low creatine levels perhaps indicate a high-scale utilization of this energy-facilitating compound for replenishing in-demand ATP for dysfunctional cells and/or an impaired internal production and intra- and extra-cellular transport of creatine (at least in CCDS). Whether low creatine in many acquired conditions is a consequence of a disease or a causative factor (or both) remains poorly addressed. However, supplemental creatine or creatine analogs appear to partially or totally restore tissue creatine levels and attenuate clinical features of many maladies, including CCDS [36,72,73,74], mitochondrial encephalomyopathy, lactic acidosis, stroke-like episodes (MELAS) syndrome [75], acute oxygen deprivation [76], chronic fatigue syndrome [77], Huntington disease [78], treatment-resistant major depressive disorder [79,80], methamphetamine-complemented depression [81], chronic heart failure [82], and rheumatoid arthritis [83], to name just a few. How much creatine needs to be compensated likely depends on the degree and type of creatine shortage; some conditions may entail covering amounts one magnitude over average dietary requirements (for a detailed review, see Ref. [84]). A vast majority of pharmacovigilance studies demonstrated favorable safety of supplemental creatine, with creatine posing no adverse health risks in humans across various life stages and conditions, at dosages ranging from 0.03 to 0.8 g per kilogram of body weight per day for up to 5 years, and dosages commonly prescribed for CCDS and other diseases/syndromes 2 to 20 g/day (for a detailed review, see Refs. [39,84]). In line with affirmative evidence from safety trials, the U.S. Food and Drug Administration (FDA) recently recognized creatine monohydrate as a safe ingredient (Generally Recognized as Safe, GRAS) [85]. This labels creatine as a non-toxic food substance under the conditions of its intended use, and perhaps expands its use across various nutritional domains. Still, determining the most bioactive chemical formulation of creatine remains a challenge [86].

## 6. Conclusions

**Figure 1:**
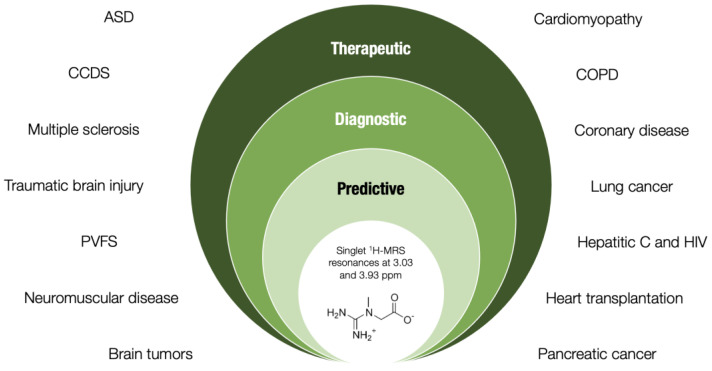
Theoretical framework of tissue creatine assessment via proton magnetic resonance spectroscopy (MRS) in clinical science. *Abbreviations*: ASD, autism spectrum disorder; CCDS, cerebral creatine deficiency syndromes; PFVS, post-viral fatigue syndrome; COPD, chronic obstructive pulmonary disease.

A drop in creatine levels accompanies various hereditary and non-hereditary diseases, and the human brain, skeletal muscle, and heart appear to be affected rather badly by creatine shortage. Being either an etiological factor or a secondary finding, lower tissue creatine concentrations almost always refer to a more severe phenotype, implying a key role of creatine in normal homeostasis and health protection. Evaluating tissue creatine (predominantly by non-invasive techniques) might thus assist in an appropriate diagnosis, prediction, and even a nutritional treatment of a specific disease characterized by creatine shortfall, with the assay thus providing an additional value in terms of health care (Figure 1). Still, to recognize tissue creatine as a potential novel biomarker candidate, additional studies are highly warranted to substantiate its biological plausibility, practicability in real-life situations, sensitivity and specificity, and cost-effectiveness, along with its responsiveness to treatment (such as exogenous creatine) in various clinical populations.
